# Applications of Three-Dimensional Printing in the Management of Complex Aortic Diseases

**DOI:** 10.1055/s-0042-1750410

**Published:** 2022-12-20

**Authors:** Paolo Magagna, Andrea Xodo, Mirko Menegolo, Carlo Campana, Luciano Ghiotto, Loris Salvador, Franco Grego

**Affiliations:** 1 Operative Unit of Cardiac Surgery, AULSS8 Berica, “San Bortolo” Hospital, Vicenza, Italy; 2 Vascular and Endovascular Surgery Division, Padova University, School of Medicine, Padova, Italy

**Keywords:** 3D Printing, 3D-printed models, aortic diseases, surgical training, preoperative planning

## Abstract

The use of three-dimensional (3D) printing is gaining considerable success in many medical fields, including surgery; however, the spread of this innovation in cardiac and vascular surgery is still limited. This article reports our pilot experience with this technology, applied as an additional tool for 20 patients treated for complex vascular or cardiac surgical diseases. We have analyzed the feasibility of a “3D printing and aortic diseases project,” which helps to obtain a more complete approach to these conditions. 3D models have been used as a resource to improve preoperative planning and simulation, both for open and endovascular procedures; furthermore, real 3D aortic models were used to develop doctor–patients communication, allowing better knowledge and awareness of their disease and of the planned surgical procedure. A 3D printing project seems feasible and applicable as an adjunctive tool in the diagnostic–therapeutic path of complex aortic diseases, with the need for future studies to verify the results.

## Introduction

Aortic disorders involve a multitude of severe clinical conditions (aortic aneurysms, acute aortic syndromes, chronic dissections, or coarctations), associated with high morbidity and mortality rates if untreated. 1 In recent years, the development of technologies and materials, together with the increased “know-how” of dedicated specialists, has improved the surgical outcomes for these diseases. 2 The creation of diagnostic–therapeutic paths dedicated to the patients suffering from an aortic disease and the latest generation image editing tools are key elements to ensure a correct surgical, hybrid, or endovascular approach, allowing more precise and accurate periprocedural planning and treatments. Even though medical imaging has become overwhelmingly more sophisticated, it is not always possible to obtain a “real vision” of the disease. A single projection (as in X-rays), a two-dimensional (2D) sequence as for magnetic resonance imaging (MRI) or for multislice computer tomography, and a 3D reconstruction obtained by surface are important methods, but sometimes they may be inadequate to widely understand a clinical situation or the critical issues of some particular cases. In such circumstances, a 3D printing solution may help to obtain a “life-like” rendering of what may be found in the operating room, optimizing the therapeutic approach and addressing complex clinical and surgical scenarios. 3 Some authors have reported their experience with patient-specific trials prior to endovascular aortic aneurysm repair (EVAR) using 3D models, reducing total procedural time, while other physicians have used 3D models to improve understanding of aortic diseases in particular for young surgeons. 4 5 In this article, we present our experience with 20 patients, treated for complex cardiac or vascular surgical diseases ( Table 3 ), for whom 3D printing has been applied as an additional tool, to improve doctor–patient communication, preoperative planning, and both endovascular and open surgical simulation of the procedures. The aim of this report is to examine the feasibility of this “3D printing and aortic disease” project to offer a constant and daily integrated additional tool for cardiac and vascular surgeons.

## Aortic Disease Project and Three-Dimensional Printing Process

The Cardiac Surgery Unit of Vicenza “San Bortolo” Hospital (Italy) led to the creation of a “3D printing and aortic diseases” project, launched 1 year ago in collaboration with the Vascular and Endovascular Surgery Clinic of the University of Padova (Italy). This project included the creation of 20 printed 3D models of the most complex aortic pathologies, over a period of 12 months. The printed products, in the present report, will be used exclusively for educational purposes. As these were not medical devices, the use was not regulated by the legislation in the EU 745/2017 (medical device regulation). Patients provided their consent to have printed product images published in this work.

The 3D models created were used to facilitate the preoperative planning of complex aortic procedures, allowing also both endovascular and open surgical training by silicone models. 3D prototypes were also used during the preoperative interview with the patients, to explain in a better and simpler way their conditions and the planned intervention.

## Three-Dimensional Model Production

**Fig. 1:**
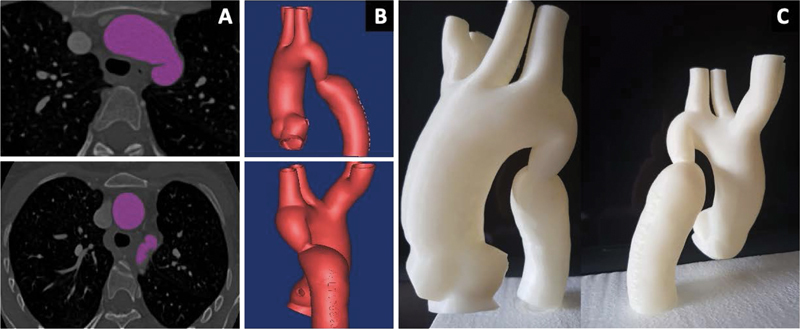
Different steps of the three-dimensional (3D) printed model production *.* ( **A** ) Axial visualization and acquisition of the region of interest, highlighted in purple. ( **B** ) Computed tomography angiography volume reconstruction. ( **C** ) 3D printed model.

The process to produce 3D-printed models consists of three main steps ( Fig. 1 , Table 2 ):

- Image acquisition.
- Image postprocessing.
- 3D printing.

### Image Acquisition

The data necessary for the 3D model production are acquired through a computed tomography angiography (CTA). The quality of the printed models depends significantly on the quality of CTA images, and a dedicated protocol has been defined for exam execution in patients with aortic disease ( Table 1 ). Cardiac motion and breathing artifacts have a negative impact on the segmentation and, thus, the printed volume; therefore, to overcome this issue, high-resolution scans are used in combination with electrocardiographic gating, breath-hold, and/or respiratory gating.

**Table 1:** Computed tomography angiogram execution technique

Parameters	Direct phase	Arterial phase with cardiac gating	Late arterial phase	Venous phase
Slice thickness	3 mm	0.4/0.6 mm	0.4/0.6 mm	1.5 mm
Extension	Thoraco-abdominal	From SATs to the computerized tomography	From computerized tomography to femoral bifurcations	From ICA to the ischial tuberosities
Contrast	−	120 cc; flow 4 cc/s	−	−

Abbreviations: ICA, internal carotid artery; SAT, supra-aortic trunks.

**Table 2:** Key steps for a three-dimensional model production

Main steps	Time required	Costs	Critical issues
Image acquisition	35 minutes	200 €	High image quality is recommended
Image post-processing	8 hours	200 €	Dedicated software; operator experience
Three-dimensional Printing	24–72 hours	1,000–1,500 €	Dedicated software and machine; operator experience; long process (represents a limitation in an urgent setting)

### Image Postprocessing

A dedicated software (Meshmixer 3.5—Autodesk, Inc., San Rafael, CA or Invesalius 3.0.0—Centro de Tecnologia da Informação Renato Archer, Campinas, SP, Brazil) is then used to obtain axial, coronal, and sagittal sections: this particular software is equipped with a “plug-in” that allows integration of diagnostic images with 3D computer-aided design (3D CAD) and computer-aided manufacturing modeling. This step identifies the ideal imaging segmentation or the ideal Digital Imaging and Communications in Medicine image sequence.

***3D reconstruction*** — ***first level:*** the software elaborates 3D surfaces, described by the outlines of what was previously selected during the segmentation phase. The obtained 3D model can be exported in a .STL format, the ideal one to interact with the 3D printer.

***3D reconstruction*** — ***second level:*** the biomodel is then corrected and refined from possible digital incongruities. In this phase, the virtual structure is “remodeled” and adapted to the use on rapid prototyping machines. The obtained file will be the definitive one, ready for printing.

### Three-Dimensional Printing Process

The final file is opened via GrabCAD Print software (9 Camp St 2nd Floor Cambridge, MA). The first step consists in repositioning the virtual object in the most suitable orientation, identifying images that offer the best compromise between the quality of the result and the layering, and evaluating both the modeling strategy and the support that should sustain the component. Once the best position is defined, the file is cleaned from possible imperfections by the automatic repair function. Subsequently, the parameters are defined starting from the choice of the material that will be used to build the model. In case acrylonitrile butadiene styrene (ABS) is chosen, the program will allow different selections.

- The automatic thickness increases when the thickness is too less.
- The layer resolution can reach 1⁄8 mm.
- The optimal type of grid used to build the support.

Once the model is obtained, with two types of nontransparent ABS (solid for the model and soluble for the support), it is treated with a “chemical bath” to complete the dissolution of every part of the support, both inside and outside of the component. Afterward, when adding the project to the print queue, the file is elaborated, displaying on the PC screen the printing preview with the expected material consumption and the process duration. The prototyping machines can be monitored remotely. It is possible to install the GrabCAD App on a smartphone, allowing the live monitoring of the last frame of the work in progress and providing information regarding nozzle temperature as well as the percentage of the work performed. At the end of the production, each prototype is verified and cataloged by the project manager. It is important to emphasize how 3D prints are highly reproducible, with an average interprint variability of less than 1 mm in all three dimensions with this machine.

## Three-Dimensional Models and Their Application for the Three-Dimensional Printing and Aortic Disease Project

### Role for Open and Endovascular Surgical Planning

In the endovascular surgery and minimally invasive cardiac surgery era, preoperative planning before a surgical procedure plays an increasingly important and crucial role. 6 These fields of surgery require different approaches to aortic and heart diseases, lacking a “de visu” approach to the target organ.

For example, regarding endovascular surgery, the choice of stent-grafts for thoracic endovascular aortic repair is usually based on CT images; however, in complex cases, such as Type B aortic dissection, it can still be considered uncertain. 3D printing enables the on-demand generation of medical devices and endografts that are unique to a specific patient. This characteristic is in sharp contrast to the current paradigm of using devices that are a rough approximation of patient anatomy (off-the-shelf devices). The fabrication of “physician modified endografts” is simplified by a 3D-printed replica model, printed from patient CTA and including aortic and main arterial branch diameters and the angle at visceral vessels origins. 7 To accommodate patient-specific branch vessel orientation, fenestrations or scallops can subsequently be added to the standard commercial stent-grafts. The added value of a 3D model in this particular scenario may be to allow faster and more accurate placement of fenestrations, reducing measurement errors; Tong et al recently reported their experience with 34 patients who underwent total endovascular repair of thoracoabdominal aortic aneurysms or dissection, using 3D-printed models to guide on-site stent-graft fenestration. This technique appeared feasible and more accurate than the traditional measurement method, with a printing process that took only 5 hours.

Open surgery as well still represents a challenge for the surgeon, as a result of the unpredictable anatomy. For example, huge aortic arch aneurysms can be treated in a single-step operation using a frozen elephant trunk. In these cases, the morphology of the landing zone is essential information for the selection of the stent-graft, making the preoperative planning difficult despite modern imaging techniques. Preoperative simulation using 3D models allows surgeons to establish the ideal endograft and its deployment, thus increasing their operative confidence.

Also, for other complex cases, such as reinterventions (redo sternotomy), open aortic arch surgery, or open Type B aortic dissection or thoracoabdominal aortic surgery, the surgeon has to control many details, requiring careful and meticulous planning. These considerations may include the cannulation site for extracorporeal circulation, the site for selective antegrade perfusion, or the aortic clamp positions during a thoracoabdominal aortic reconstruction.

Although open surgical repair remains the gold standard approach for many diseases, both in cardiac and vascular surgery, minimally invasive techniques have grown in popularity and continue to evolve. Between July 2013 and September 2018, 125 patients underwent isolated aortic valve replacement using an endoscopic surgical approach at “San Bortolo” Cardiac Surgery Department. 8 This technique requires proper training and should be done by surgeons highly experienced in minimally invasive and endoscopic surgery. 3D resin models were inserted inside the prosthetic thoracoscopic training box to simulate the approach to the ascending aorta. In 10 cases, personalized 3D-printed models with realistic anatomical characteristics were used to perform more accurate planning, especially in cases of chronic aortic dissection, aortic arch aneurysm, and aortic coarctation.

**Fig. 2:**
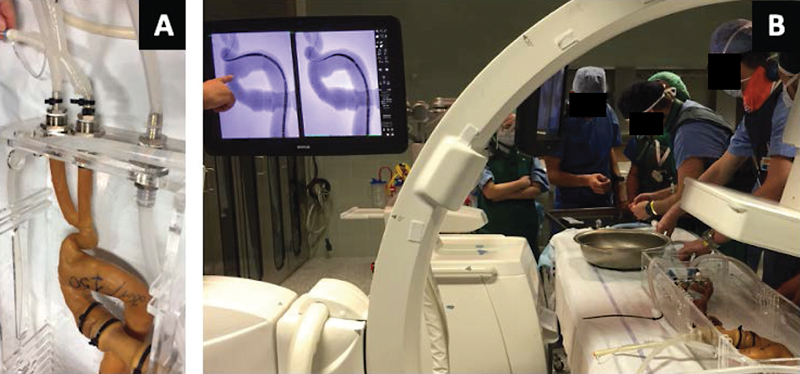
Silicone three-dimensional (3D) printed model, used to perform a simulation before a real surgical procedure in a patient suffering from an aortic arch aneurysm *.* ( **A** ) The 3D model is connected to a pulsatile flow system to simulate the antegrade flow. ( **B** ) Surgical simulation phase in the operation room.

At the Padova Vascular and Endovascular Surgery Division, starting from the printed model, a silicone 3D simulation has been produced and connected to a pulsatile flow system in three complex endovascular aortic cases, to perform an endovascular surgical simulation before the real procedure. Regarding the case represented in Fig. 2 , the patient suffered from an aortic arch aneurysm, excluded from open surgical repair due to his comorbidity. The flow system allows the team to simulate the navigability of the devices, performing an “on bench” procedure with an antegrade flow.

As pointed out in current published studies, there can be savings in operating time when using 3D-printed models prior to surgery. 9

### Role for Young Surgeons Training

Open surgical procedural skills are essential prerequisites that a surgeon should develop before advancing to more technically demanding operations such as endoscopic or endovascular techniques. Although minimally invasive procedures offer lower morbidity and mortality, vascular surgeons should combine skills in both open and endovascular treatments and open vascular surgery should be considered the “starting point” for the endovascular surgery learning process. Also for cardiac surgery, a thoracoscopic approach must begin from a deep knowledge of traditional cardiac surgery.

Simulation-based training is obviously not a substitute for clinical practice; however, the utility of 3D-printed models promotes surgical skills acquisition outside the operating theater, providing opportunities for intensive training. Surgical anatomy is concerned with 3D structures. The increased number of endovascular or minimally invasive procedures may limit the “familiarity” of young surgeons with traditional open surgical repair, in particular for complex cases such as the reinterventions. 10

An option for cardiac and vascular surgeon training is cadaveric simulation, although this approach is expensive and often limited by ethical issues. 11 Endovascular virtual simulation has gained considerable importance, demonstrating a high level of reproducibility and relevance to reality. 12

**Fig. 3:**
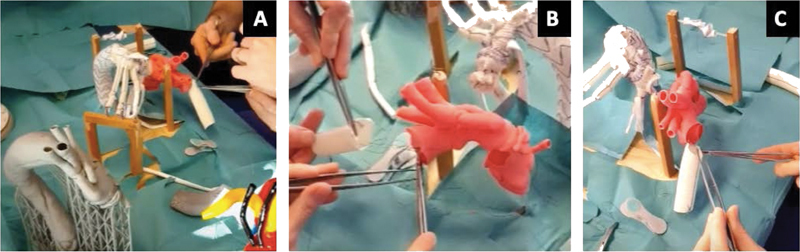
( **A–C** ) Some phases of the “bench” surgical training on three-dimensional models, identical to the real anatomy of the patient.

Fig. 3 shows some phases of the “bench” surgical training of young surgeons, who under the supervision of consultants perform anastomoses on 3D models, anatomically identical to the real structures of the patient. These structures are produced with a particular resin (Everes Functional Model Sisma SpA, Vicenza, Italy), deformable and hollow, allowing physical simulation of the aorta and aortic branches, as well as clamping and vascular anastomosis.

Endovascular procedures represent a radically new way of operating compared with open surgical repair. For example, during EVAR, the surgeon deals with a closed abdomen, with the only information about vascular anatomy obtained from the preoperative angiogram (CTA) and the intraoperative angiographic images: the surgeon has no direct contact with the aorta, its branches, the aneurysm, or the surrounding structures. In this scenario, 3D models can be an important tool for planning, especially for young surgeons who are approaching endovascular procedures. In fact, thanks to 3D models, it is possible to better understand the patient's anatomy in the preoperative phase, accurately identifying the aortic landing zones, the most appropriate endograft, and its length and the oversizing rate. 13 A possible reduction of the procedure time in the operating room thanks to the simulation may also be possible allowing reduction in radiation exposure of patient and staff.

### Roles in Doctor–Patients Communication

Guidance General Medical Council in the UK strongly advocates a collaborative approach by physicians with their patients. 14 In particular in cardiac and vascular surgery, the therapeutic choice between an endovascular or mini-invasive procedure and an open procedure must be shared with the patient, referring him to a center with high experience in both methods; understanding the clinical status, prognosis, and possible therapies is essential for a patient to participate in treatment. The patient's knowledge of his condition is easier to foster in the presence of a 3D model, which allows to “touch” the disease and to better understand the surgery to treat it, increasing trust with the surgeon.

The 3D model could also be used by involving the family doctor during the informed consent explanation; because of their long-term relationships with patients and sensitivity to psychosocial issues, family physicians can engage patients in collaborative health care decision-making. 15

Communication is vital for doctors and patients and the complex nature of heart, and vascular diseases of surgical interest, such as dissections or aneurysms or congenital heart disease, are particularly good examples of this. There is an “expert to nonexpert” interaction, with little likelihood of a patient's full awareness of the disease. This state of uncertainty about the health condition and treatment increases the chances of unsuccessful communication. Patient-specific 3D models offer a potential new means to improve communication in this challenging environment, improve parental understanding, and possibly reduce the stress of consultation. 16

### Limitations

Despite the potential of 3D printing, there are several hurdles to overcome. The cost of this additive technology is relevant because of the availability of machines and software to create the models and the time to plan and produce the models, without delaying surgical procedures.

The time phases for the printing of the models include several steps, from the execution of the CTA to the data transmission time to the printing laboratory for the segmentation, up to the real printing time of the 3D model. From the CTA to the final model production, the overall time required is approximately 72 to 96 hours, which can be reduced to 48 to 72 hours in cases of urgency; time also depends on the materials used (resins, silicones, and ABS) and on the extension of the pathology and, therefore, the size of the model. Regarding this project, the use of 3D printing was limited to elective cases, in particular, due to manufacturing time reasons.

Meanwhile, the cost for a prototype can be quantified as approximately 1,200 to 1,700 euros; the costs are aligned with those of other similar studies and among the current limitations of this project, it should be emphasized that the Italian Public Health System, nowadays, does not provide reimbursements for medical 3D models (and also for other important applications such as virtual/augmented/mixed reality), making these projects usually limited to research centers or university hospitals. 17

To spread these methods, the contribution of research funds or private donations is, therefore, very important today and studies like this could help to incentivize political administrations to introduce public incentives or reimbursement forms for 3D printing.

Another limitation may be the absence of an accepted process to assess the accuracy of 3D models, especially regarding the models used in the simulation of endovascular or cardiac surgery, especially to ensure mechanical properties similar to human tissue. The reproducibility of the models is instead ensured with an average interprinting variability of less than 1 mm for the ABS reconstructions: we have no experience with the use of other types of printers than the one used for the project.

**Fig. 4:**
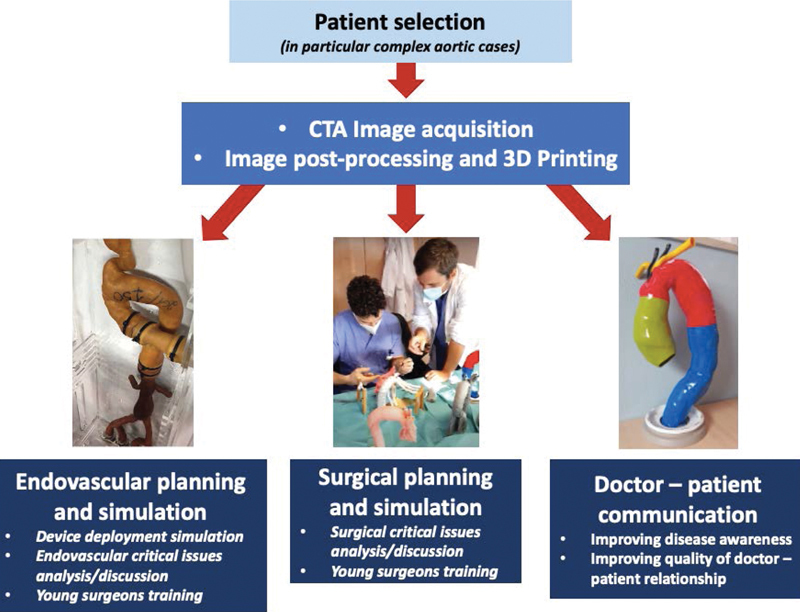
A flow chart summarizes the production steps and the different uses of the three-dimensional models.

Despite all these limitations, in our opinion the future potential of this method is considerable in various sectors: the “transversal” applicability of this project, in our little experience, has been summarized in a flow chart ( Fig. 4 ).

### Future Applications

Future advances are likely to result in improved manufacturing time and cost of 3D printers, as well as a greater variety of raw materials to fabricate the models. To improve the availability of 3D-printed models, a standardized protocol may be necessary for the creation of the models on a large scale and with short manufacturing time. Such a protocol should include technical details, such as the 3D printer model used, the software, the print material, and degree of resolution. These steps may be the key to establishing further the cost-effectiveness of 3D printing and its ability to improve patient outcomes. The use of a questionnaire to evaluate the impact of 3D models on the doctor–patient relationship before surgery will be evaluated at our institution in the near future. 18 Despite significant technological advances in tissue engineering, there are still challenges for the development of tissue constructs that mimic their natural counterparts. Bioprinting has emerged as a technology that would create highly organized 3D vascular networks, within engineered tissue constructs, to promote the transport of oxygen, nutrients, and waste products, which can hardly be made using conventional microfabrication techniques.

Aortic tissue models were created using cell aggregates of mouse embryonic fibroblasts combined with a 3D bioprinted support structure. 19 Experts in this field believe that a radical improvement in tissue engineering may derive from 3D printing. 20

## Conclusion

This report demonstrates our preliminary experience in creating a pathway for patient-specific 3D models of different cardiovascular surgical diseases. The application of 3D printing provides an additional tool to the surgeon, to address the complexity of heart and aortic pathology, both from a strictly surgical point of view and as regards the doctor–patient relationship. 3D models can enhance in preoperative planning for complex cases, while also providing a tool for surgical training on models nearby identical to the real anatomy. 3D printing may come to have a large influence on vascular and heart surgery, proving especially important for the next generation of surgeons and health care professionals. Future studies to verify the results are needed.

**Table 3:** Clinical and anatomical details of the conditions of the 20 patients for whom a three-dimensional model was produced

Disease	Number of patients	Max aortic diameter (mm)	Previous sternotomy/laparotomy	Reason of three-dimensional printing
Aortic root pseudoaneurysm	3	62	Yes	Retrosternal adhesion (pulmonary artery—RV)
Aortic coarctation	2	37	Yes	Endovascular planning
AoArch pseudoaneurysm	2	58	Yes	Endovascular planning
AsAo pseudoaneurysm	4	68	Yes	Retrosternal adhesion (pulmonary artery—RV)
Aortic valve regurgitation + AoArch pseudoaneurysm	2	64	Yes	Retrosternal adhesion (pulmonary artery—RV); SATs anatomy
Infrarenal AAA	1	75	No	Accessory renal artery—OSR planning
Juxtarenal AAA	1	66	No	ICV anatomical variant—OSR planning
TAAA (II Type)	4	72	No	Endovascular planning
TBAD + aberrant RSA	1	49	No	OSR planning

Abbreviations: AAA, abdominal aortic aneurysms; AoArch, aortic arch; AsAo, ascending aorta; OSR, open surgical repair; RSA, right subclavian arteries; RV, right ventricle; TAAA, thoracoabdominal aortic aneurysms; TBAD, Type B aortic dissection.
